# Visual Iconicity Across Sign Languages: Large-Scale Automated Video Analysis of Iconic Articulators and Locations

**DOI:** 10.3389/fpsyg.2018.00725

**Published:** 2018-05-15

**Authors:** Robert Östling, Carl Börstell, Servane Courtaux

**Affiliations:** ^1^Department of Linguistics, Stockholm University, Stockholm, Sweden; ^2^Centre for Language Studies, Radboud University, Nijmegen, Netherlands; ^3^École Nationale Supérieure de Techniques Avancées, ParisTech, Paris, France

**Keywords:** iconicity, sign language, location, two-handed signs, semantics, lexical plurality, automated video processing, typology

## Abstract

We use automatic processing of 120,000 sign videos in 31 different sign languages to show a cross-linguistic pattern for two types of iconic form–meaning relationships in the visual modality. First, we demonstrate that the degree of inherent plurality of concepts, based on individual ratings by non-signers, strongly correlates with the number of hands used in the sign forms encoding the same concepts across sign languages. Second, we show that certain concepts are iconically articulated around specific parts of the body, as predicted by the associational intuitions by non-signers. The implications of our results are both theoretical and methodological. With regard to theoretical implications, we corroborate previous research by demonstrating and quantifying, using a much larger material than previously available, the iconic nature of languages in the visual modality. As for the methodological implications, we show how automatic methods are, in fact, useful for performing large-scale analysis of sign language data, to a high level of accuracy, as indicated by our manual error analysis.

## 1. Introduction

### 1.1. Iconicity across languages and modalities

The traditional view of the linguistic lexical unit has been that it is created by combining meaningless units (phonemes) into a meaning-bearing form (morpheme/word) which is semantically not compositional or even motivated, i.e., it is arbitrary (de Saussure, 1916). Arbitrariness of word forms has even been used as a criterion for what constitutes *language* (Hockett, 1960). However, the view of the building blocks of language as being entirely arbitrary has later been questioned after it has been found—across languages—that both units smaller than words and words themselves may exhibit non-arbitrariness. The clearest case of non-arbitrariness is *iconicity*, the direct form–meaning association in which the linguistic sign resembles the denoted referent in form, which has been found across languages in both the spoken and the signed modality (Perniss et al., 2010; Meir and Tkachman, 2018). However, it is often claimed that signed languages are more iconic than spoken languages, because the former are—due to the visual modality—“richer in iconic devices” (Meir and Tkachman, 2018).

For spoken languages, sensory imagery appears to be particularly associated with various forms of iconic sound symbolism (e.g., *splash, beep*) (e.g., Dingemanse, 2012; Schmidtke et al., 2014; Kwon and Round, 2015; Dingemanse et al., 2016; Winter et al., 2017), and has also been recreated in experimental settings (e.g., Köhler, 1929; Lockwood and Dingemanse, 2015; Cuskley et al., 2017; Fort et al., 2018). Urban (2011) and Blasi et al. (2016) demonstrate that there are a number of iconic form–meaning mappings across large samples of the world's spoken languages, such as the mapping between phonemes and its associated articulatory body part (e.g., /n/ with “nose,” /l/ with “tongue,” and bilabials with “lip”), or the mapping between phonemes and physical properties (e.g., /i/ with “small”). Although iconic expression is often concrete, it may also be extended to abstract senses (Auracher, 2017). Thus, iconicity seems to be an integral part of language (Dingemanse et al., 2015), which has been found to be a facilitating element when acquiring a language, regardless of the modality (e.g., Thompson et al., 2012; Monaghan et al., 2014; Lockwood et al., 2016; Ortega, 2017). In their overview of research on (non-)arbitrariness in the vocabularies of human languages, Dingemanse et al. (2015) conclude that there are trade-offs in making use of arbitrariness and non-arbitrariness (e.g., iconicity). Whereas arbitrariness offers fewer constraints on form, it makes it harder for users to learn; and whereas iconicity facilitates learning, it may restrict the forms and abstraction possibilities of the language. We hope that our present work in the visual modality can contribute toward determining the parameters involved in this trade-off.

The type of iconicity is possible is partly dependent on the modality. For example, whereas quantity (size, plurality, intensity) is easy to depict iconically with either spoken or signed forms (e.g., by duration or reduplication), spoken language is better for depicting sound, and signed language is better for depicting visual properties and space (Dingemanse et al., 2015, 608)^1^. In this paper, we are mainly interested in the visual iconicity found among signed languages.

### 1.2. Sign language iconicity

Already some of the earliest research on signed languages acknowledged the iconic motivation found in many signs (Klima and Bellugi, 1979). That is, the form of a sign is directly motivated by visual properties of its referent. This is a quite uncontroversial claim today, and there is a growing body of work looking at the interaction between iconicity and the structure of signed language (e.g., Taub, 2001; Meir, 2010; Lepic, 2015). However, it has been argued that there is a language-dependent factor in the iconicity of signs, such that signers tend to rate the signs of their own language as more iconic than the signs with corresponding meanings in another sign language (Occhino et al., 2017). Those looking at iconicity as a factor shaping the very structure of signed languages have noted that individual form features of a lexical sign can provide separate parts of the combined semantics of the whole. For instance, the handshape may be used to describe size and shape properties of an entity, by letting the hand represent either the entity itself, or the hand as it handles the entity (Padden et al., 2013). Similarly, the type of movement in the articulation of a sign may be motivated, such as having movement manner, duration, and onset/offset encode lexical aspect iconically, such as distinct end movements being associated with telicity (Grose et al., 2007). In a model developed by Taub (2001), the form and meaning of signs can be formalized as a so-called *double mapping* in which form parameters are mapped to the concrete source of a metaphor, which in turn are mapped onto a metaphorical target. Using this model, Meir (2010) shows how the Israeli Sign Language sign for “learning” makes use of the metaphor understanding is grasping. In this sign, the handshape represents holding an object, which is interpreted metaphorically as considering an idea, and the sign location (forehead) represents the head, which is metaphorically linked to the location of the mind^2^.

In this paper, we are specifically interested in two broad phonological parameters of signs: number of hands and sign location. Both of these parameters have been found to contribute to the iconicity of signs.

Any sign can be produced with either *one or two hands*. This dichotomy has initially mostly been treated merely as a phonological feature of signs (van der Hulst, 1996), perhaps because the distribution of one- and two-handed signs across sign language lexicons seems to be 50/50 (Börstell et al., 2016; Crasborn and Sáfár, 2016), suggesting a random distribution^3^. However, it has been shown that the number of hands used in a sign can be attributed to meaning, based on the iconic mapping between the articulators (e.g., the hands) and (parts of) a referent, for example as plural/reciprocal alternations (Pfau and Steinbach, 2003, 2006, 2016) or multiple entities (e.g., Dudis, 2004; Zwitserlood et al., 2012). However, the use of two hands for plural expression has also been shown to work on the lexical level. For example, Lepic et al. (2016) showed that while the distribution between one- and two-handed signs in any random sign language lexicon appears balanced and arbitrary, there is significant overlap in which meanings are encoded by two-handed signs *across* languages. Using what the authors term *articulatory plurality*, Börstell et al. (2016) argue that sign languages are able to map plural referents onto the plural articulators. Börstell et al. (2016) and Lepic et al. (2016) show that sign languages favor two-handed sign forms across languages to represent lexically plural concepts. Lexical plurals are concepts that carry some form of inherent plurality in their semantics, for example *reciprocals*, events/relationships necessarily involving multiple participants (“kiss,” “argue,” “friend”) (Haspelmath, 2007; Acquaviva, 2008), and *mass/dual/plural groups or objects*, involving multiple members/parts (“army,” “socks,” “gloves”) (Koptjevskaja-Tamm, 2004; Acquaviva, 2008, 2016; Wisniewski, 2010; Lauwers and Lammert, 2016; Mihatsch, 2016). The association between plurality and two-handed forms is argued to be an iconic mapping in the same domain as the association between repeated or longer word forms and quantity/plurality (Dingemanse et al., 2015), based on the metaphor more of form is more of content (Lakoff and Johnson, 1980, p. 127).

Another parameter of the lexical sign is its *location* (or, *place of articulation*), which means the place in signing space, on or around the signer's own body, at which a sign is produced. A sign location may be lexically specified or modified. For example, any lexical sign has a lexically specified location—e.g., the signs eat and say are typically signed at the mouth, in an iconic fashion (Frishberg and Gough, 2000; Taub, 2001). However, the location may sometimes be altered in order to indicate associations with referents localized in signing space (Cormier et al., 2015; Occhino and Wilcox, 2016). Since the lexical location may be iconic (e.g., eat at the mouth), there may be restrictions to the possible modifications of locations in a sign. For example, if the location is iconic, modifying the location results in a loss of (metaphorical) iconic mapping which may be disallowed by the language (Meir, 2010; Meir et al., 2013). In this paper, however, we focus on the issue of location only as a lexically specified parameter of a sign, in order to evaluate quantitatively, within and across sign languages, to what extent this parameter is iconically motivated. Location has long been known to constitute a possible iconic parameter of lexical signs. Locations may be directly or metaphorically associated with a certain meaning—i.e., locations may be iconic. For instance, the forehead is associated with cognition, whereas the chest is associated with emotion (Brennan, 1990, 2005). Although this is a well-known property of lexical locations, it has never been quantified to any larger extent. An attempt at quantifying the form–meaning mapping of sign locations was made by Börstell and Östling (2017) by linking the manually annotated Swedish Sign Language dictionary to a semantic dictionary. This showed that signs of certain semantic domains (e.g., “think,” “see,” “eat”) were more prominent in certain locations (forehead, eyes, and mouth/belly, respectively) than signs in general.

In this paper, we aim to investigate the *number of hands* and *location* in signs and their association with specific semantics. We do this using automated methods on a large dataset with 120,000 videos from a sample of 31 different sign languages, using the parallel sign language dictionary *Spread the Sign* 2012,^4^. We specifically hypothesize that (1) the iconic mapping strategy of articulatory plurality, specifically using two-handed sign forms to represent plural meanings, is employed across languages, and (2) sensory and body part-related meanings will be iconically articulated at their associated locations on the body across languages.

## 2. Materials and methods

We use data from the online parallel dictionary *Spread the Sign* 2012, which contains a total of 31 sign languages with sign entries, and roughly 300,000 videos of individual signs. Table 1 shows the sign languages included in our dataset. The language sample is not typologically balanced, showing a clear bias toward European or European-derived sign languages. The issue of genealogical relatedness and contact is notoriously difficult when it comes to sign languages. There is little research on the topic, and most classifications are based on either historical sources of contact (usually concerning deaf education) or lexicostatistical comparisons classifying languages based on lexical similarity (cf. Brentari, 2010; Jepsen et al., 2015). A basic attempt at a classification is found under the *Sign Language* family in the *Glottolog* database, according to which several of the languages in our dataset are categorized as part the same language group (e.g., Swedish Sign Language and Finnish Sign Language, or German Sign Language and Polish Sign Language) (Hammarström et al., 2017). In this study, we are not concerned with the potential relatedness between languages. The aim is to explore iconic properties of any language in the visual modality and the possible patterns that may be discerned. However, it should be noted that some patterns of metaphorical iconicity found here may be influenced by having a European/Western-biased language sample.

**Table 1:** The 31 sign languages in the dataset.

American Sign Language	Estonian Sign Language	Latvian Sign Language
Austrian Sign Language	Finnish Sign Language	Lithuanian Sign Language
Belarusian Sign Language	French Sign Language	Polish Sign Language
Brazilian Sign Language	German Sign Language	Portuguese Sign Language
British Sign Language	Greek Sign Language	Romanian Sign Language
Bulgarian Sign Language	Icelandic Sign Language	Russian Sign Language
Chinese Sign Language	Indian Sign Language	Spanish Sign Language
Croatian Sign Language	International Sign	Swedish Sign Language
Cuban Sign Language	Italian Sign Language	Turkish Sign Language
Czech Sign Language	Japanese Sign Language	Ugandan Sign Language
		Ukrainian Sign Language

### 2.1. Aims

In this study, we use computer vision techniques to infer the main areas of hand activity for individual sign videos. This allows us to study visual iconicity in sign languages in several ways: by comparing patterns of articulation and iconicity between different semantic concepts and categories within the same language, or by comparing signs for the same concept or in the same semantic category across different languages in order to see possible patterns across the sign languages of our sample. Our two main research questions are the following:

1. What is the distribution of one- vs. two-handed signs across sign languages?
  - Is the general distribution of one- vs. two-handed signs 50/50, when looking at a semantically diverse set of items, as found by Lepic et al. (2016) and Börstell et al. (2016)?
  - Is lexical plurality associated with two-handed signs, as argued by Börstell et al. (2016)?
2. Is there a cross-linguistic pattern of sign locations being iconically associated with specific meanings, as found by Börstell and Östling (2017) for Swedish Sign Language (e.g., “think” associated with the forehead)?

We approach these two main research questions in two studies. Study 1 deals with identifying the number of articulators (i.e., one- vs. two-handed signs) and correlating this with plurality. Study 2 deals with the visualization of sign location based on hand activity and the correlation with semantics. Thus, the two studies aim to show the extent to which visual iconicity is found across sign languages with regard to articulators and locations. The studies also provide us with the possibility of evaluating how signed language can be analyzed with the help of automated video processing methods. By utilizing such methods, we are able to quantify some of the claims about sign language iconicity previously investigated with much smaller datasets in terms of the number of languages and the number of signs involved.

### 2.2. Data processing

Video files were downloaded from the *Spread the Sign* public website, along with metadata on the language used, as well as the name of the concept and the concept's category (in English)^5^. As the first step in our processing chain, we used the body pose estimation model of Cao et al. (2016) to identify the position of wrists and elbows for each video frame. We used the model file published by the authors, which has been trained on the COCO dataset (Lin et al., 2014)^6^. While their model can identify the poses of multiple humans in a frame and thus is much more general than needed here, we found that it is highly accurate in identifying the required body parts. This step required about two months of computing time on a single GPU.

**Figure 1:**
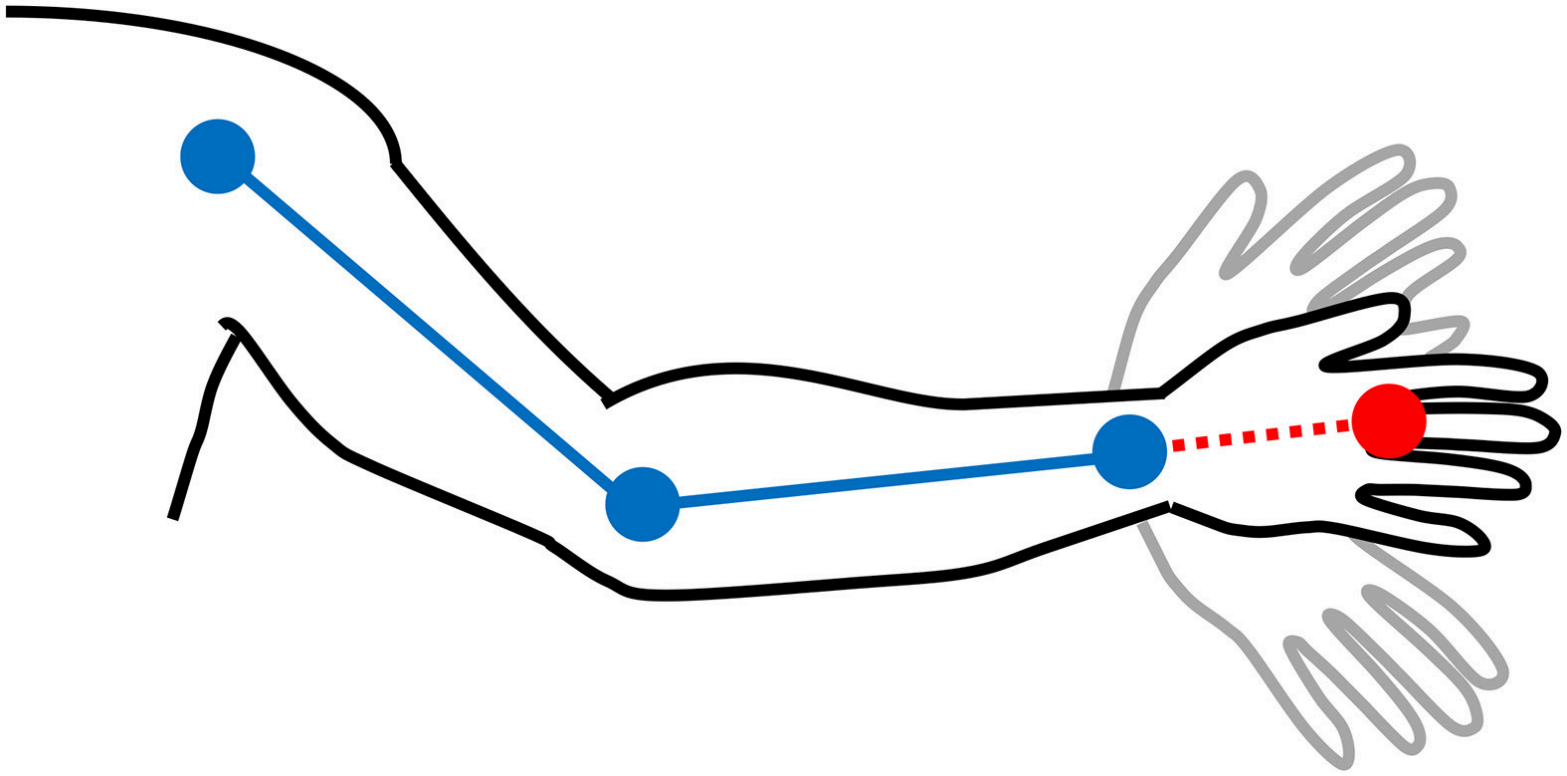
The hand location (red) is extrapolated based on the automatically detected joints (blue) by adding half the distance between the elbow and wrist to the wrist location as a straight line from the elbow and wrist joints (dotted red line).

**Figure 2:**
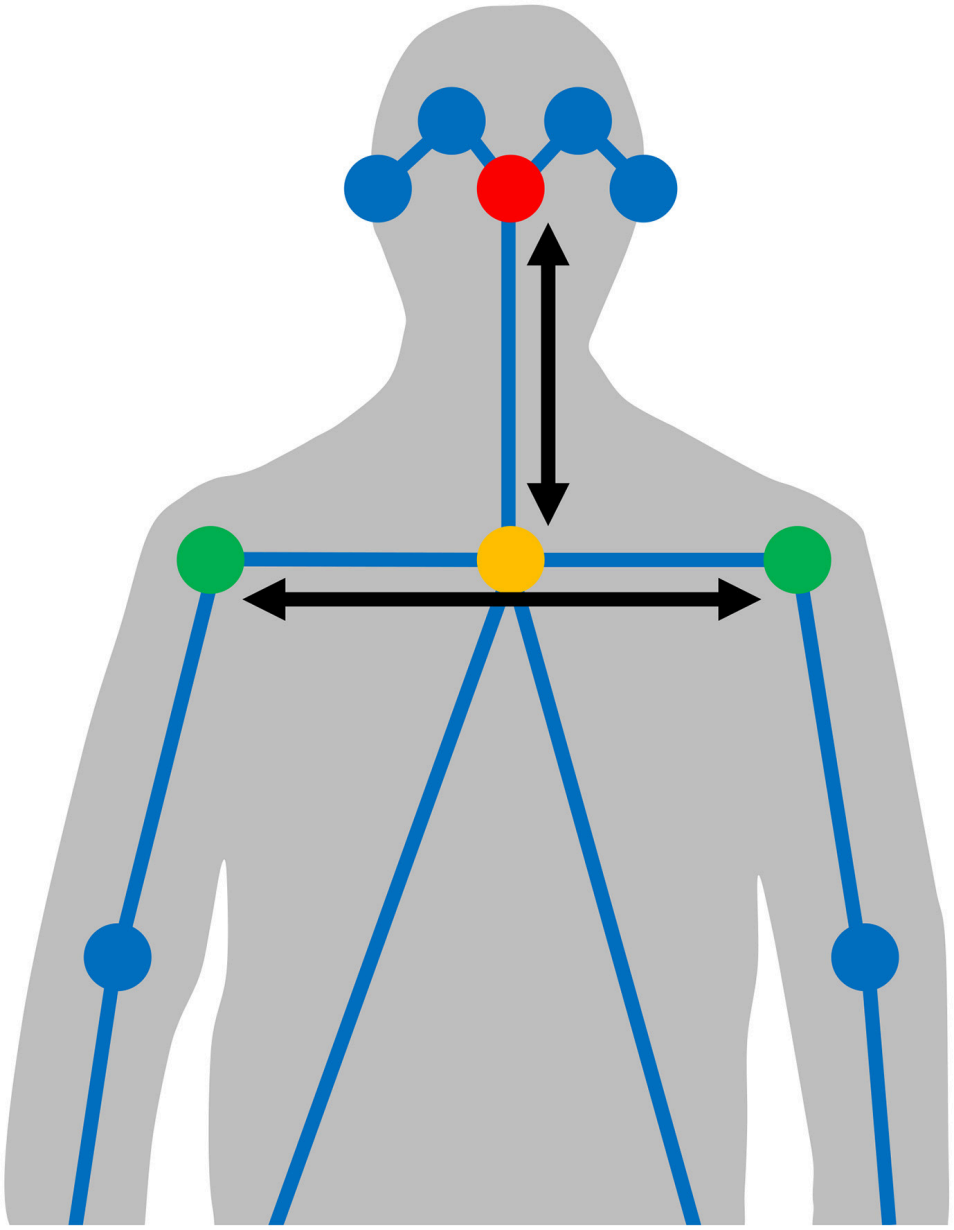
The automatically detected joints (circles) and connectors (lines) identified by the body pose estimation model. Signer body normalization is done based on the averaged location of the signer's nose (red circle) as origo, after which the *x* axis is scaled by the mean distance between the shoulders (green circles), and the *y* axis is scaled by the mean distance between the nose and the neck (yellow circle).

Since the body pose estimation model is not directly trained to detect hands, we estimate their location by extrapolating the elbow–wrist line outwards by half the distance between the elbow and wrist. As shown in Figure 1, this assumes a hand position based on a straight wrist joint and the lower knuckles (the metacarpophalangeal joints) as the center of the hand, which is a fairly close approximation in most cases, but discards any possible wrist flexion. Furthermore, since there is considerable variation in body shape and camera distance, we normalize the coordinates such that the averaged location of the signer's nose is at origo, the *x* axis is scaled by the mean distance between the shoulders, and the *y* axis is scaled by the mean distance between the nose and the neck (Figure 2). This normalization is performed per video.

### 2.3. Study 1: number of articulators

Our main interest is to explain *why* certain signs are two-handed while others are not. In particular, based on previous studies (Börstell et al., 2016; Lepic et al., 2016) we expect that lexical plurality is an important semantic component in predicting whether a sign is two-handed or not. Although lexical plurality has been researched extensively across many languages (e.g., Acquaviva, 2008, 2016; Lauwers and Lammert, 2016), we include here data from a plurality rating task, in order to account for plurality—or, specifically the *perceived* plurality—of concepts as a scalar property^7^. For this, we designed a questionnaire as described in section 2.3.2 below. Another possible explanation that we explore is the influence of frequency, since the principle of economy suggests that high-frequency concepts should have forms that are less costly to articulate, which in turn could influence the number of articulators used for specific signs (cf. Crasborn and Sáfár, 2016, p. 244). For this, we used the lemma frequency of the concept's English name in the British National Corpus (Leech et al., 2001).

#### 2.3.1. Data processing

We estimate the number of articulators by calculating the total length of the path traced by each hand (in the normalized coordinate system) during a sign. If the path length of one hand exceeds the other's by a factor of more than 3, we classify the sign as one-handed. All other signs are classified as two-handed. In rare cases, the video processing stage fails to identify the hand(s). To deal with this we discard all signs with hands detected in <10 frames.

#### 2.3.2. Plurality rating questionnaire

Since little quantitative data exists on lexical plurality, we sent out an online questionnaire and collected plurality ratings from respondents (*N* = 23; 10 female, 13 male; mean age 29, *SD* 10), mainly those without a linguistics background. Respondents were asked to rate the *lexical plurality* of 100 concepts, 50 of which were taken from a previous study (Börstell et al., 2016), collected from various sources (Attarde, 2007; Haspelmath, 2007; Wisniewski, 2010) identifying them as lexically plural concepts, and 50 which were random concepts frequency matched (pairwise) to the lexically plural concepts in another study (Börstell et al., 2016). Lexical plurality is defined in the questionnaire as “whether or not there is some inherent plural meaning of the concept (e.g., involving multiple parts/participants/events).” Concepts were presented in random order, and respondents were asked to provide ratings on a discrete scale from 1 (“not at all plural”) to 7 (“definitely plural”). For our analysis, 19 concepts from the questionnaire were excluded for one of two reasons:

1. 5 concepts: Body parts for which Börstell et al. (2016) used a plural form (e.g., “eyes”) while *Spread the Sign* uses a singular form (e.g., “eye”).
2. 14 concepts: Missing data from *Spread the Sign*.

Thus, a total of 81 concepts with both plurality ratings and number of hands in *Spread the Sign* remained to be used in our analysis.

#### 2.3.3. Statistical model

We model our data in the following way. The plurality rating of respondent *i* for concept *c*, *R*_*c,i*_, is assumed to be an independent draw from a normal distribution with standard deviation σ^*Q*^ and mean (1 + 6*p*_*c*_) + *r*_*i*_, that is, $R_{c,i}\sim N\left(1+6p_{c}+r_{i},\sigma^{Q}\right)$. Note that the unobserved ‘true’ plurality *p*_*c*_ is a continuous variable on the interval [0, 1] while *R*_*c,i*_ ∈ {1, 2, 3, 4, 5, 6, 7}. To account for individual bias among respondents, we add an individual noise term $r_{i}\sim N\left(0,\sigma^{R}\right)$ for respondent *i*. We assume a logistic regression model for the probability of a sign *S*_*c,l*_, expressing concept *c* in language *l*, being two-handed: $P\left(S_{c,l}=1\right)=\sigma \left(\beta_{p}p_{c}+\beta_{f}f_{c}+\alpha_{l}^{L}+\alpha_{c}^{C}+a\right)$, where the logistic function σ(*x*) = 1/(1 + *e*^−*x*^). The unobserved variables of the model are listed in Table 2, and the data are summarized in Table 3. Weakly informative priors are used throughout, since we do not have strong prior knowledge to further constrain the model. Logistic regression coefficients are unlikely to have absolute values much above 5, so we use *N*(0, 5) priors. Standard deviations of either concept/language-specific regression terms or rating scores are also unlikely to be much above 5, so we use exponential priors with λ = 1/5. For inference, we used the NUTS sampler implemented in the Stan software package (Carpenter et al., 2017) to run four independent chains with 5,000 burn-in iterations followed by 5,000 sampling iterations. All parameters have the Gelman-Rubin statistic $\hat{R}<1.01$, indicating convergence.

**Table 2:** Unobserved variables in our statistical model.

**Variable**	**Prior**	**Description**
β_*p*_	*N*(0, 5)	Plurality coefficient
β_*f*_	*N*(0, 5)	Frequency coefficient
*p*_*c*_	*U*(0, 1)	Plurality of concept *c*
*r*_*i*_	*N*(0, σ^*R*^)	Effect of rater *i* on rating
$\alpha_{l}^{L}$	*N*(0, σ^*L*^)	Effect of language *l* on number of hands
$\alpha_{c}^{C}$	*N*(0, σ^*C*^)	Effect of concept *c* on number of hands
*a*	*N*(0, 5)	Intercept term
σ^*Q*^	Exp(1/5)	Standard deviation of *R*_*c*, ·_
σ^*R*^	Exp(1/5)	Standard deviation of α^*R*^
σ^*L*^	Exp(1/5)	Standard deviation of α^*L*^
σ^*C*^	Exp(1/5)	Standard deviation of α^*C*^

**Table 3:** Observed variables in our statistical model.

**Variable**	**Description**
*R*_*c,i*_	Plurality rating (integer 1–7) of concept *c* from respondent *i*
*S*_*c,l*_	Number of hands (1 = two, 0 = one) used in the sign expressing concept *c* in language *l*
*f*_*c*_	log-frequency in British National Corpus of concept *c*, scaled to [0, 1]

### 2.4. Study 2: sign locations

For visualizing articulator activity over a large number of signs, we also use the normalized coordinates of the hands. These are used to trace the path of each sign individually on a grid. The path is then blurred to reflect the uncertainty in our estimate of the hand location, by convolving it with a Gaussian function (σ = 0.03125*x*, where *x* is the horizontal resolution of the figure), and placed on top of a silhouette positioned as a reference point in relation to the positions of the body pose joint coordinates (see Figure 2). The values on the grid are then averaged over the group of signs to be visualized together. In our visualizations we color the activity of the left hand in blue and the right hand in red. The final strength of each color in the visualizations represents the mean amount of time, across languages, each hand spends at a certain location.

#### 2.4.1. Quantifying iconicity through location ratings

**Figure 3:**
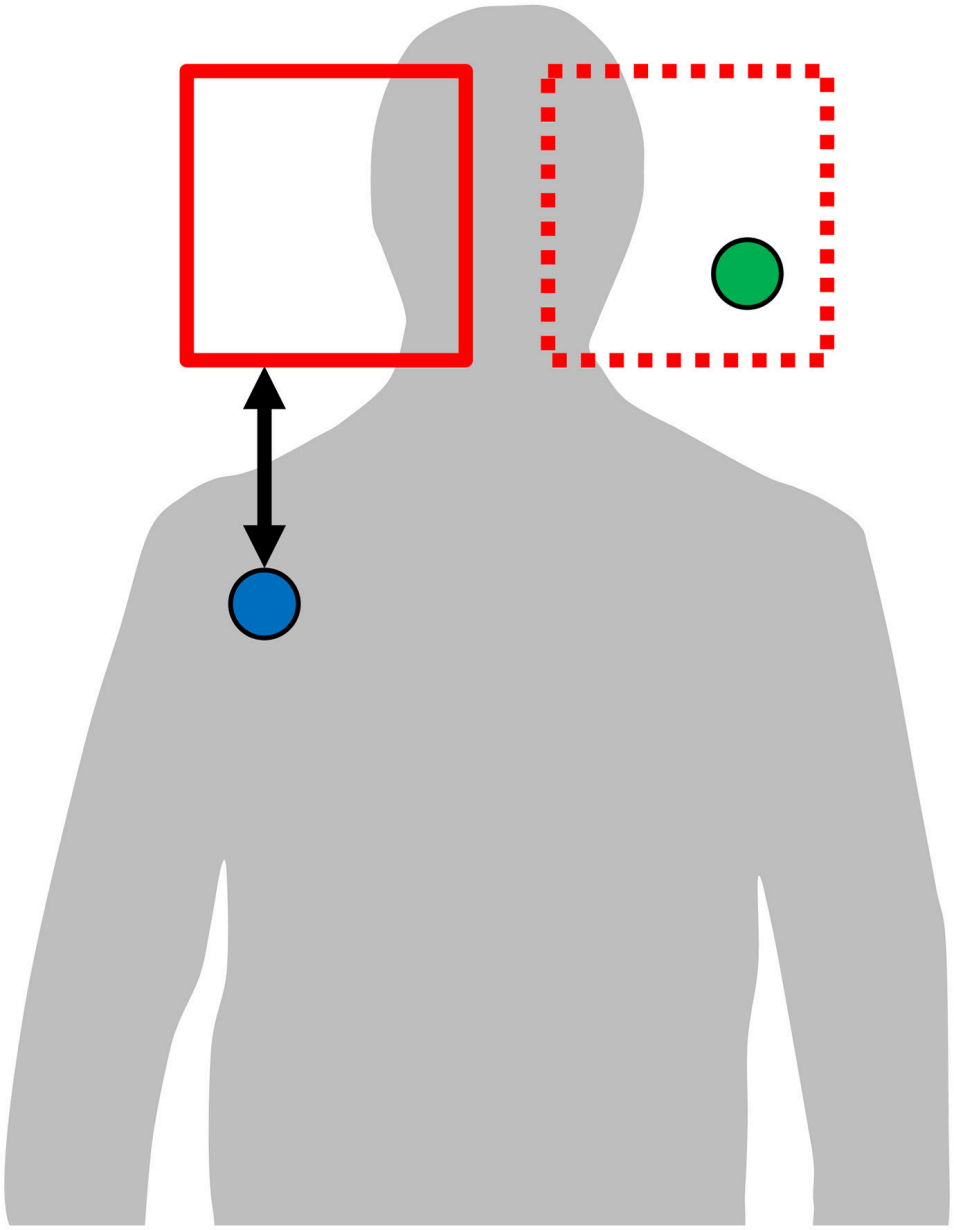
Respondents place a rectangle (red) on the part of the silhouette they judge most iconic for a concept. The rectangle's area is mirrored symmetrically (dotted red). Hand coordinates falling within either original or mirrored rectangle (green circle) receive the distance 0. Hand coordinates outside the rectangles (blue circle) receive the distance to the nearest rectangle border (black arrow).

To demonstrate that systematicity in sign locations across languages is in fact attributed to iconicity, we define a quantitative measure of iconicity. To obtain data, we created a computer-based visual questionnaire for *location ratings*. Respondents (*N* = 10; 6 female, 4 male; mean age 41, *SD* 13), none of which reported knowledge of any sign language, were instructed to place a rectangle on a body silhouette (see Figure 3). This is the same silhouette we use for visualization of the hand activity across concepts. Respondents were presented, in random order, with the name of the concept in English and the silhouette, and were instructed to place a single rectangle—the size of which could be controlled by the respondent—on the part of the image that they most strongly associate with the concept. To ensure symmetry, the rectangle is mirrored so that any part covered in the left half of the body is also covered on the right half, and vice versa. We refer to the resulting rectangle(s), either one or two, as a *location rating*. The *iconicity score* of a sign with respect to a location rating is then computed in the following way:

1. Guess the dominant hand by choosing the hand with the longest trajectory length during the sign. In case of symmetric two-handed signs, the choice of dominant hand will be arbitrary, but since the computations are symmetric this will not affect the result.
2. All video frames without the dominant hand are removed, and then the first 20% and the last 20% of remaining frames are removed, because they tend to include movements from and to a neutral position.
3. The *iconicity score* is now computed as the negative mean distance, over all remaining video frames, between the dominant hand and the closest rectangle in the location rating. If the hand is inside a rectangle, the distance is zero for that frame. Otherwise, Euclidean distance between the hand and the closest point of any rectangle is used (see Figure 3). Thus, the highest possible iconicity score (zero) is obtained if the whole sign is articulated *entirely* inside the space marked by *all* respondents in the location ratings. Lower values (that is, increasingly negative) of the iconicity score indicate increasing divergence form the location ratings.

To compute the iconicity score for a concept, we compute the *N* × *M* iconicity scores for each combination of the *N* languages that have a sign for the concept and the *M* location ratings for that concept, and use the mean of these values. Thus, the higher the iconicity score, the closer the articulation of the signs are to the areas indicated by the respondents.

The iconicity score is difficult to interpret out of context. For this reason, we also compare the location ratings to each concept in a vocabulary list, to estimate the level of chance similarity. We use the Swadesh list from Study 1 (see section 2.3), with the concept food added in order to ensure that all the concepts in this study are covered. If iconicity is a significant factor, we expect the location ratings of a particular concept to be closer (have a higher iconicity score) to signs expressing the same concept, than to unrelated signs.

## 3. Results

### 3.1. Study 1: number of articulators

#### 3.1.1. Processing quality

To assess the soundness of our method, we decided to manually annotate 20 randomly sampled signs for each language: 10 that were classified as one-handed, and 10 that were classified as two-handed^8^. All the sampled concepts are in the Swadesh list. With this information, we are able to estimate the precision for each category (one-handed and two-handed). While statistical power is limited due to the small sample, we can easily identify two languages for which the automatic processing fails completely: Czech Sign Language and Russian Sign Language. For these languages, only 3 of the 10 signs classified as two-handed are in fact two-handed. For two other languages, British Sign Language and Portuguese Sign Language, 7 of 10 signs classified as two-handed are really two-handed. All other languages contained at most 2 errors per group of 10 for two-handed sign detection, and all languages (including the ones above) have at most 2 errors per group of 10 for one-handed sign detection. Qualitatively, we found that these errors were mainly due to camera setup in these languages, in which the non-articulating hand was partly in frame (and moving slightly) during one-handed signs, thereby confusing the body pose estimation model. Overall precision for one-handed sign detection is 95.0% (13 errors in 260 signs), and 95.8% (11 errors in 260 signs) for two-handed sign detection if the four problematic cases mentioned above are removed. Without removing these, precision for two-handed sign detection drops to 89.7% (31 errors in 300 signs) while one-handed sign detection remains high at 95.7% (13 errors in 300 signs). We take this as a proof of validity for our automated method in identifying the number of hands in sign videos.

#### 3.1.2. Distribution of one- vs. two-handed signs

Table 4 shows the distribution of one- and two-handed signs in each language, for both *core vocabulary* and *extended vocabulary*. We define the *core vocabulary* to include all signs available in the data that represent concepts in the Swadesh list from Lepic et al. (2016), in total 5,667 signs for 195 concepts. Going beyond the core vocabulary is problematic, since two-handedness becomes a less meaningful property when dealing with compound signs or whole phrases, and *Spread the Sign* does not contain enough information to distinguish these from simple signs. In order to obtain an *extended vocabulary* of mostly non-compound signs, we use all signs for concepts with single-word translations in an isolating language (English). This results in 122,935 signs, an amount that would be very time-consuming to classify manually, hence our automatic processing demonstrates its usefulness.

**Table 4:** Number of one-handed (**1H**) and two-handed (**2H**) signs according to the *Spread the Sign* data for concepts in the Swadesh list (**Core vocabulary**) and for all concepts with a single-word English translation (**Extended vocabulary**).

	**Core vocabulary**			**Extended vocabulary**		
**Sign language**	**1H**	**2H**	**2H/(1H+2H) (%)**	**1H**	**2H**	**2H/(1H+2H) (%)**
American Sign Language	102	130	56.0	2,216	3,535	61.5
Austrian Sign Language	100	133	57.1	1,527	3,942	72.1
Belarusian Sign Language	56	89	61.4	738	3,003	80.3
Brazilian Sign Language	126	83	39.7	1,302	1,715	56.8
^*^British Sign Language	64	170	72.6	715	4,829	87.1
Bulgarian Sign Language	85	67	44.1	588	841	58.9
Chinese Sign Language	83	93	52.8	876	2,977	77.3
Croatian Sign Language	30	39	56.5	402	919	69.6
Cuban Sign Language	6	9	60.0	22	34	60.7
^*^Czech Sign Language	23	210	90.1	248	5,260	95.5
Estonian Sign Language	100	136	57.6	1,468	4,097	73.6
Finnish Sign Language	17	12	41.4	121	92	43.2
French Sign Language	82	139	62.9	1,184	3,833	76.4
German Sign Language	109	122	52.8	1,815	3,711	67.2
Greek Sign Language	12	25	67.6	483	950	66.3
Icelandic Sign Language	118	114	49.1	1,847	3,754	67.0
Indian Sign Language	91	98	51.9	626	1,714	73.2
International Sign	39	50	56.2	185	291	61.1
Italian Sign Language	102	132	56.4	1,136	4,450	79.7
Japanese Sign Language	79	116	59.5	660	2,033	75.5
Latvian Sign Language	100	134	57.3	1,720	3,905	69.4
Lithuanian Sign Language	117	113	49.1	1,921	3,572	65.0
Polish Sign Language	103	120	53.8	1,545	3,799	71.1
^*^Portuguese Sign Language	106	117	52.5	1,527	3,669	70.6
Romanian Sign Language	96	91	48.7	1,943	1,941	50.0
^*^Russian Sign Language	71	156	68.7	1,446	3,980	73.4
Spanish Sign Language	121	110	47.6	1,670	3,759	69.2
Swedish Sign Language	109	115	51.3	1,924	3,582	65.1
Turkish Sign Language	113	117	50.9	1,976	3,412	63.3
Ugandan Sign Language	15	19	55.9	75	135	64.3
Ukrainian Sign Language	118	115	49.4	1,739	3,556	67.2
Total	2,493	3,174	56.0	35,645	87,290	71.0

*The proportion of two-handed signs [**2H/(1H**+**2H)**] is also given. Languages marked with asterisk have systematic processing errors*.

**Figure 4:**
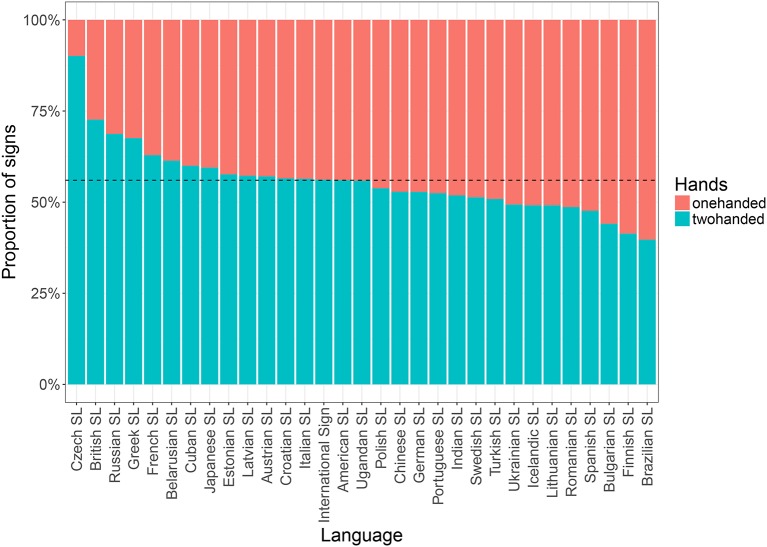
Distribution of one- vs. two-handed signs (*n* = 5, 667) across languages for concepts (*n* = 195) in the Swadesh list; dotted line showing the mean proportion of two-handed signs across all signs (56.0%). Note that all languages in our data are included here, i.e., also those excluded from our analysis due to unreliable video processing (Czech SL, British SL, Russian SL, and Portuguese SL).

**Figure 5:**
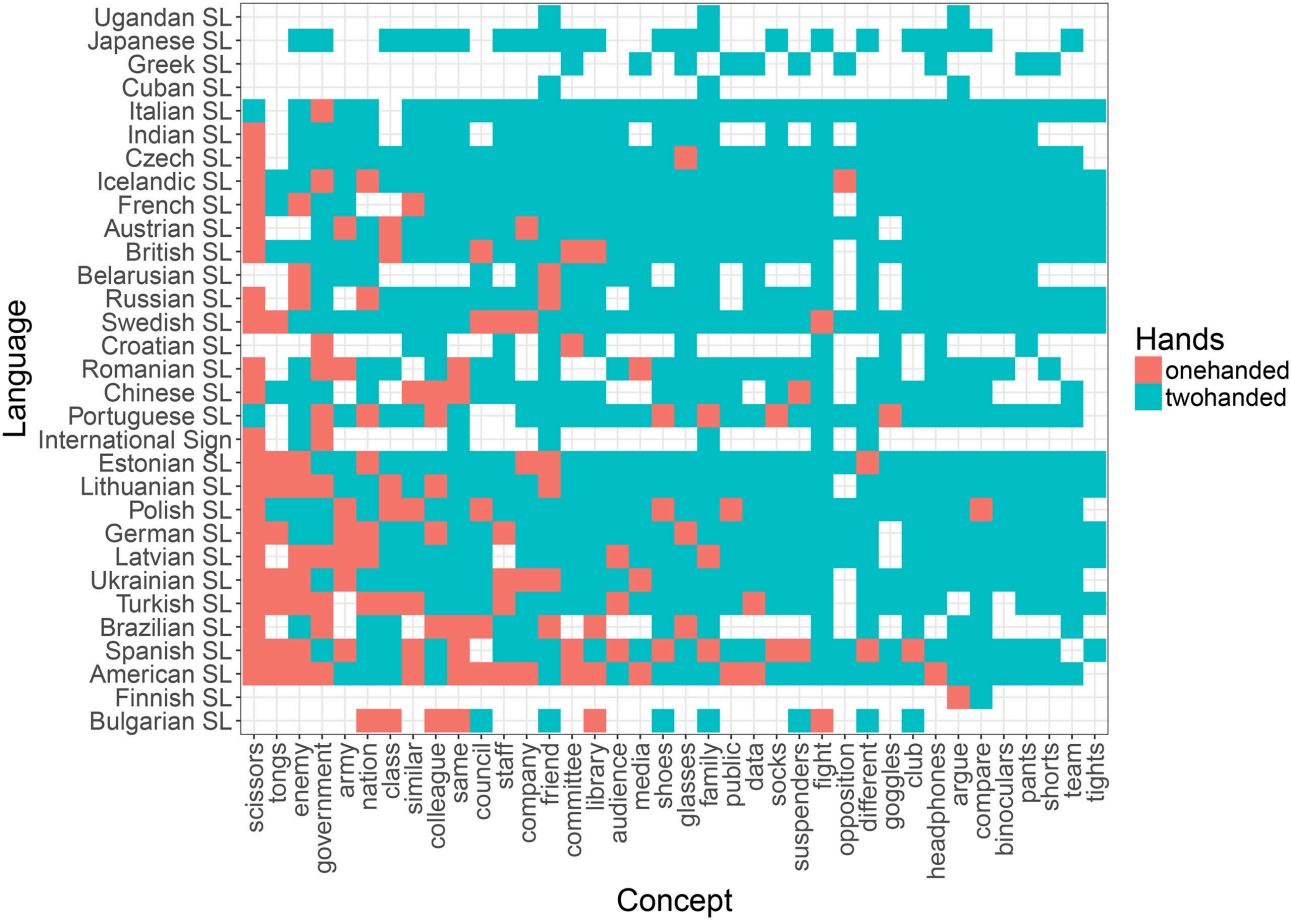
Distribution of one- (red) vs. two-handed (blue) signs across concepts (*x* axis) and languages (*y* axis) in the lexical plural list.

From these results, we see that the proportion of two-handed signs in the extended vocabulary (71.0%) is much higher than the corresponding figure for the core vocabulary (56.0%, cf. Figure 4), although still lower than in the list of lexical plurals (81.8%, cf. Figure 5)^9^. This may be explained by lexical frequency and articulatory economy. For instance, Crasborn and Sáfár (2016) show that the while the distribution of one- vs. two-handed signs in Sign Language of the Netherlands is more or less balanced in lexical databases, there is a bias toward one-handed signs in corpus tokens. The authors suggest that ease of articulation may cause lexically two-handed signs to be produced as one-handed signs (Crasborn and Sáfár, 2016, p. 244). In fact, it may even be possible that frequency effects on phonetic reduction pushes toward one-handed articulation with frequent signs, similarly to how frequent signs have been shown to be the most reduced in terms of sign duration (Börstell et al., 2016). However, seeing as other sign databases of similar size to *Spread the Sign* (per individual language) have been shown previously to exhibit a near 50/50 distribution of one- vs. two-handed signs (Börstell et al., 2016, p. 393), it is also possible that the selection of concepts in the *Spread the Sign* project affects the distribution. As stated on their website, one objective of the project was to facilitate vocational training exchanges between countries, and possibly the inclusion of vocational school terminology results in a higher proportion of complex concepts that require compound, phrasal, and/or depicting constructions, which are more likely to be encoded (in part) by two-handed forms.

#### 3.1.3. The influence of plurality

**Figure 6:**
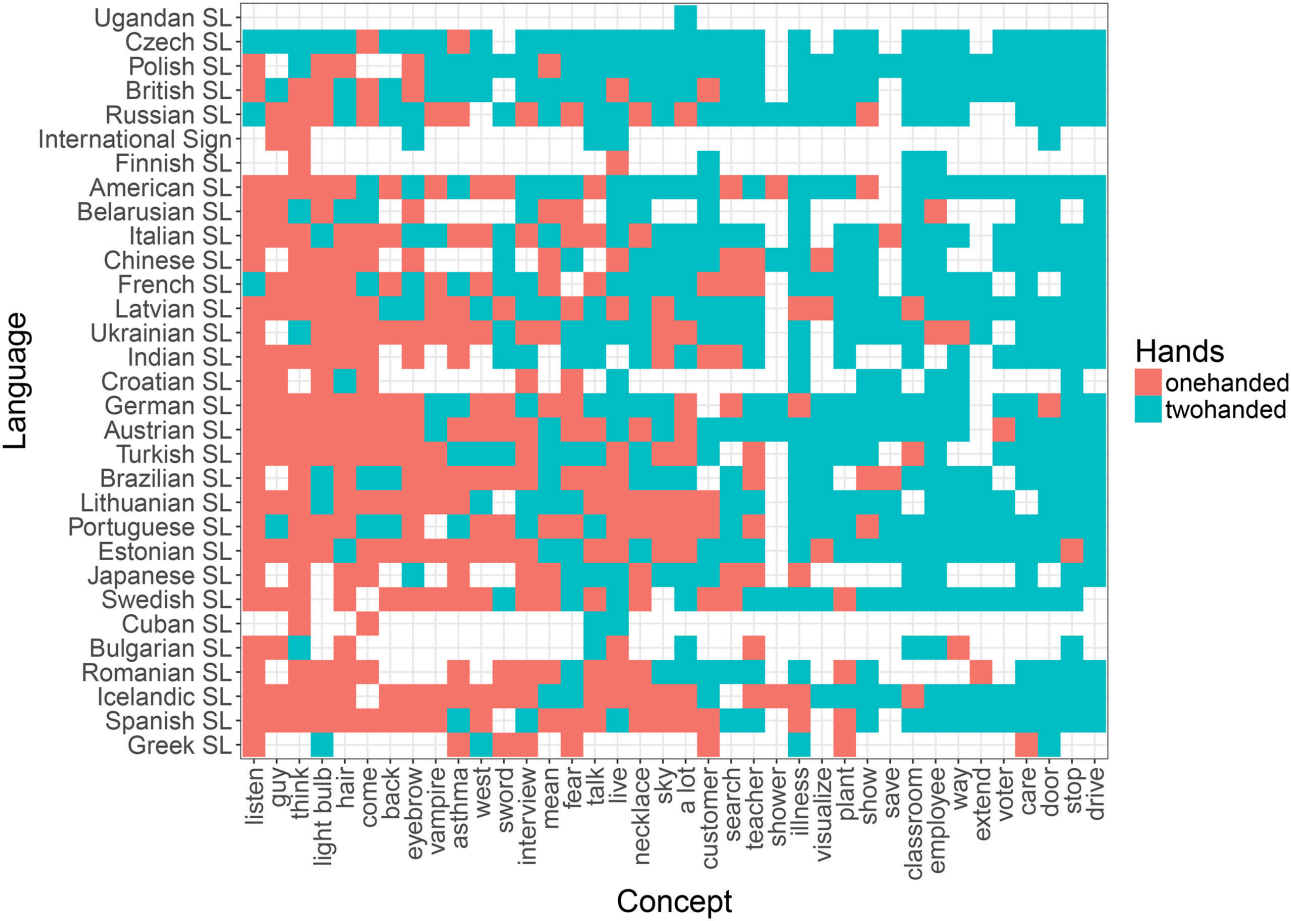
Distribution of one- (red) vs. two-handed (blue) signs across concepts (*x* axis) and languages (*y* axis) in the random list.

Based on previous research (Börstell et al., 2016) and the results obtained here (see Table 4 and Figure 4), we expect the—at least core—vocabulary of any sign language to exhibit a close to 50/50 distribution between one- and two-handed signs. As shown by Börstell et al. (2016) for a sample of 10 sign languages across five language groups, this distribution becomes heavily skewed toward two-handed signs when looking specifically at a list of lexically plural concepts (i.e., concepts that are inherently plural). The motivation for this is argued to be that sign languages make use of *articulatory plurality*, which means that they map plural referents onto the plural articulators (e.g., the two hands) in an iconic manner. An informal glance at the distribution across languages reveals that while the list of random concepts shows a quite even distribution of one- vs. two-handed signs (Figure 6), the sampled lexically plural concepts show a two-handed preference across languages (Figure 5).

**Figure 7:**
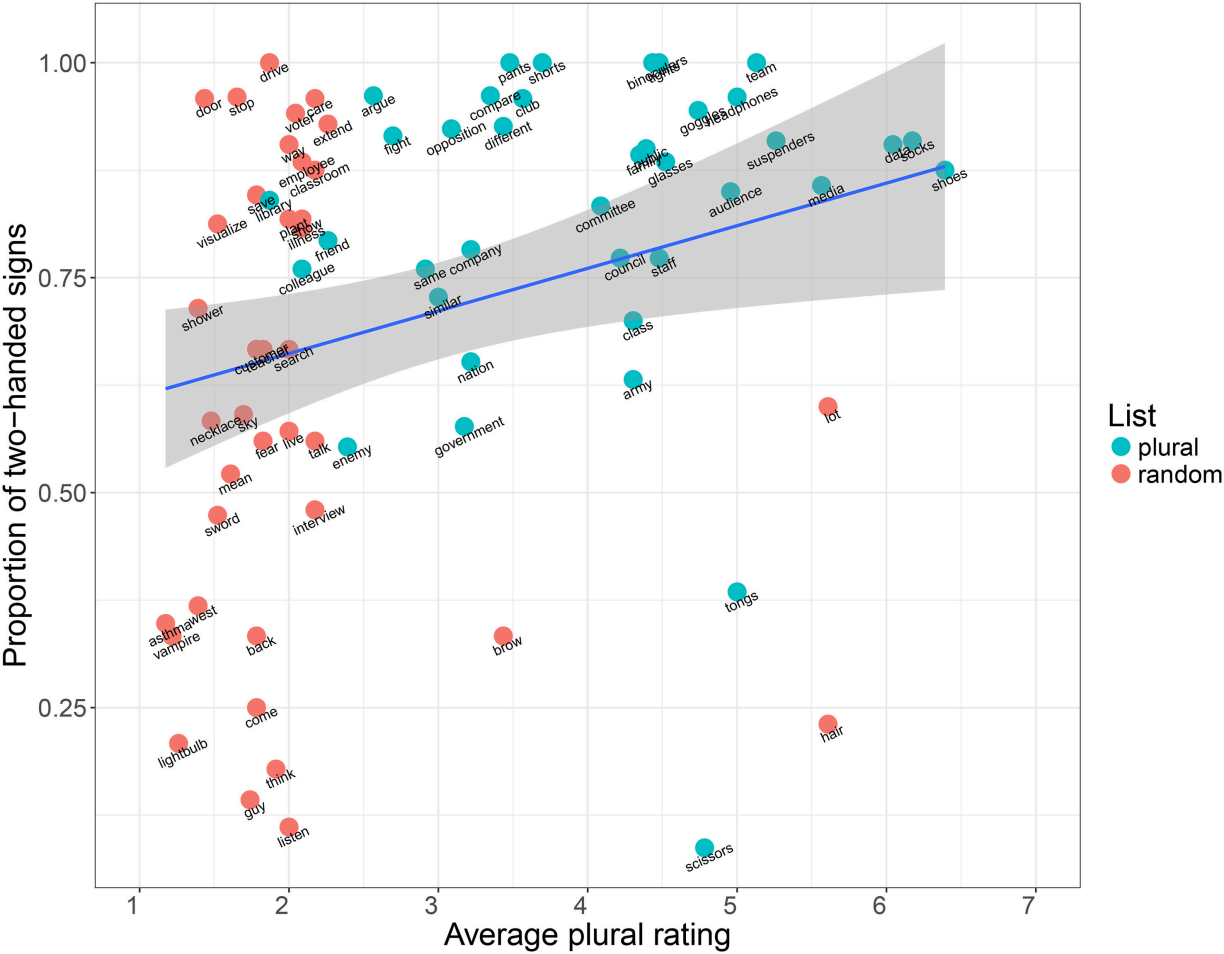
Correlation between plurality ratings (*x* axis) and the proportion of two-handed signs across languages (*y* axis) for both plural (blue) and random (red) items.

Setting the categorization of plural vs. random list aside, we also want to compare the proportion of two-handed signs to the perceived plurality of the individual concepts (section 2.3.2). Figure 7 shows the correlation between the plural ratings for individual concepts and the proportion of two-handed signs encoding the same concepts across languages. Here we see that there is a clear difference in patterning between plural and random items. Random items have generally low plural ratings (as expected), and are also evenly distributed across the *y* axis, demonstrating that non-plural items exhibit the expected 50/50 split between one- and two-handed signs. However, the plural items have mixed plural ratings, although overall much higher than the random items, and are all clearly biased toward two-handed sign forms across languages. This gives us a general visualization of plurality and two-handed forms being correlated. We have investigated this more rigorously using the model described in section 2.3.3. Its parameters were estimated using the 81 concepts in the list ranked for lexical plurality described in section 2.3.2. Table 5 summarizes some important parameters and their estimates. Due to the relatively low number of concepts studied, the posterior distributions of these parameters are fairly wide, but still allow us to draw a number of conclusions conditioned on the assumptions of our model:

- *P*(β_*p*_ > 0) ≈ 99.5% and *P*(β_*p*_ > 1) ≈ 93.0%: lexical plurality is extremely likely to be a predictor of two-handed signs, and the effect is likely to be large.
- *P*(β_*f*_ < 0) ≈ 41.7% and *P*(|β_*f*_| > 1) ≈ 26.5%: we do not have evidence for frequency being a predictor of one-handed signs, but even a large effect (in either direction) can not be excluded.
- *P*(σ^*L*^ < 0.67) ≈ 97.5%: languages are fairly consistent in the overall distribution of one- and two-handed signs, and most variation is explained by other factors.
- *P*(σ^*C*^ > 1.30) ≈ 97.5%: concept-specific properties beyond lexical plurality are important predictors of one- or two-handedness. The $\alpha_{c}^{C}$ values at the bottom of Table 5 provide some examples.

**Table 5:** Estimates of some interesting model parameters, with credibility intervals centered at the median.

**Parameter**	**Description**	**Percentile**				
		**2.5**	**25**	**50**	**75**	**97.5**
β_*p*_	Plurality regression coefficient	0.58	1.68	2.25	2.84	3.93
β_*f*_	Frequency regression coefficient	–1.58	–0.40	0.18	0.77	1.92
*a*	Intercept	–1.02	–0.15	0.29	0.73	1.61
σ^*C*^	Per-concept regression term *SD*	1.30	1.50	1.61	1.74	2.03
σ^*L*^	Per-language regression term *SD*	0.20	0.33	0.41	0.49	0.67
σ^*R*^	Per-rater bias term *SD*	0.62	0.75	0.83	0.93	1.17
σ^*Q*^	Rating noise *SD*	1.47	1.50	1.52	1.53	1.57
$\alpha_{\text{SCISSORS}}^{C}$	Regression term of concept scissors	–5.50	–4.36	–3.82	–3.32	–2.43
$\alpha_{\text{HAIR}}^{C}$	Regression term of concept hair	–5.02	–4.09	–3.63	–3.19	–2.41
$\alpha_{\text{GUY}}^{C}$	Regression term of concept guy	–5.50	–4.04	–3.39	–2.81	–1.85
…						
$\alpha_{\text{DOOR}}^{C}$	Regression term of concept door	0.50	1.41	1.93	2.49	3.75
$\alpha_{\text{SHORTS}}^{C}$	Regression term of concept shorts	0.22	1.33	1.98	2.71	4.38
$\alpha_{\text{DRIVE}}^{C}$	Regression term of concept drive	0.81	1.84	2.45	3.13	4.73

Looking at the individual concepts in the lexical plural list, we observe that all but two (“tongs” and “scissors”) are preferentially encoded as two-handed signs across languages (Figure 5). This can not be explained by any of the other factors in our statistical model (see above, and Table 5). This is also reflected in the results of Börstell et al. (2016), for which “tongs” and “scissors” were the only concepts of those overlapping with this current study that were not encoded as two-handed signs in any sign language. A manual check in the *Spread the Sign* videos shows that “tongs” are mostly referred to by a handling depiction (i.e., showing how a hand uses tongs), whereas *all* languages have a one-handed depiction of “scissors” with the fingers representing the shears. As argued by Börstell et al. (2016), using dual/plural fingers for mapping plural referents is also an instance of articulatory plurality, only using a different individuation of articulators (i.e., fingers instead of hands) in the iconic mapping between articulators and plural referents. In Figure 5, we notice that there has been a misclassification of “scissors” as two-handed for two languages (Italian Sign Language and Portuguese Sign Language).

There are also some clear examples of the opposite tendency, where the other factors in our model are unable to explain two-handedness. For instance, the concepts drive and door are nearly universally two-handed signs in our sample, in spite of receiving low plurality rankings. They both have iconic motivations, but concept-specific ones not captured by the notion of lexical plurality: driving a vehicle by holding the steering wheel (usually symmetrically, using both hands), and opening a door in a wall (asymmetric, with the non-dominant hand representing a reference point in the wall/door post). These are, however, motivations found by Lepic et al. (2016), for instance in having spatial configurations with one hand as the reference point to the other, such as in door, or using plural hands to represent plural hands, as in drive, although the meaning of “drive” is not inherently plural in itself (the plurality is in the limbs used for the activity).

### 3.2. Study 2: sign locations

For our second research question, we wanted to investigate whether there are cross-linguistically valid patterns of sign locations being iconic. Here we define iconicity based on the location ratings made by hearing non-signers asked to map concepts onto a body silhouette (see section 2.4.1). Using these ratings, we can also quantify the extent to which the cross-linguistic hand activity patterns align with non-signers' associations between concepts and the human body. Table 6 shows that out of the six concepts from Börstell and Östling (2017), four are in the 4th percentile or lower, indicating a high degree of similarity to the location ratings. The remaining two concepts, food and say, are both articulated at the mouth in nearly all of the languages in our sample. Thus, for these individual concepts, we find some clear examples of iconic locations across languages.

**Table 6:** Concepts and their similarity to location ratings (0 would indicate perfect agreement).

	**Concept only**		**All signs**	
**Concept**	**Mean sim**.	**Percentile**	**Mean sim**.	***SD***
food	–0.84	26	–0.95	0.18
hear	–0.42	3	–1.03	0.32
hungry	–0.37	1	–0.93	0.30
love	–0.28	3	–0.52	0.18
say	–0.88	32	–1.02	0.28
think	–0.49	4	–1.10	0.33

*Mean similarity scores are presented for signs in different languages representing the given concept (left) and for all signs in the Swadesh list (right)*.

**Figure 8:**
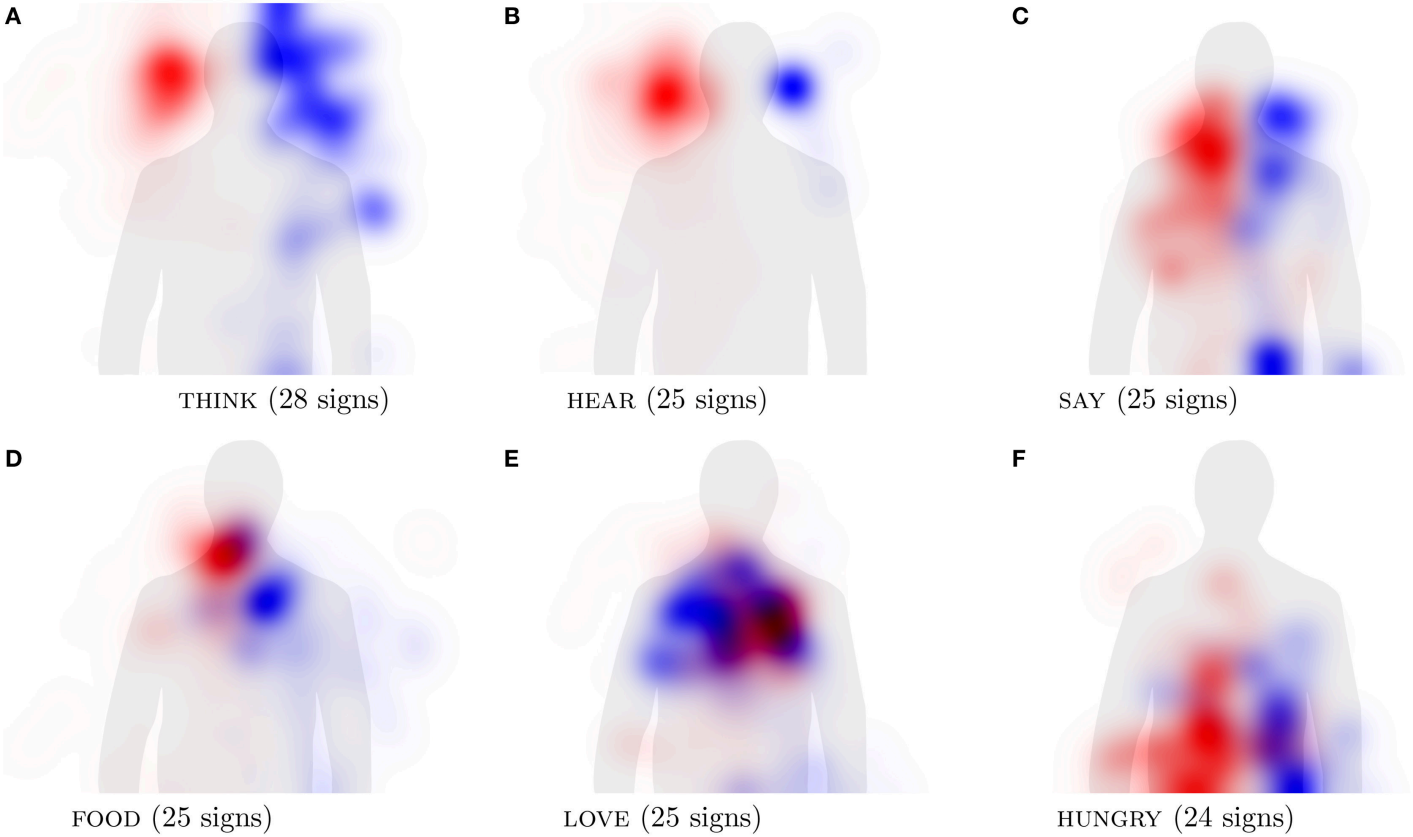
Visualization of hand activity in signs representing individual concepts across multiple languages. The activity of the right hand is shown in red and the left hand in blue.

In order to visualize cross-linguistic similarity, Figure 8 shows the hand activity across the languages in the *Spread the Sign* dictionary for the individual concepts. These concepts, also compared to the iconicity of locations task, have been chosen in part because they provide prototypical examples of the categories investigated by Börstell and Östling (2017) for Swedish Sign Language. As expected, they show strong tendencies to be located in certain areas: think (forehead), hear (ears), say and food (lower face), love (chest, with crossed arms), hungry (belly). These sign locations are clearly iconic in some sense, either directly or metaphorically: whereas “think,” “hear,” and “say” are all located at the body part directly involved in the respective activity (head/brain for thinking, ears for hearing, and mouth/throat for saying), “food” is located at the body part associated with a related action (mouth for eating). The concept “hungry” is located at the belly, in which hunger is felt, and “love” is located at the chest, which is explained by the metaphorical association of heart as the center of experiencing the emotion. In the visualization, some cases are clearer than others in that there is less cross-linguistic variation. For instance, the concept “hear” is clearly associated with the ears across languages, as is “think” with the (fore)head and “food” with the mouth. The concept “say” shows a slightly more variable location across languages, yet mostly centered around the mouth (see Figure 8C), as has previously been argued by Frishberg and Gough (2000, p. 117–118). This shows that iconic mappings between sign location and meaning—directly or metaphorically—are visible across languages, which is supported by the fact that they correlate with the locations identified as iconic for each concept by hearing non-signers (Table 6).

**Figure 9:**
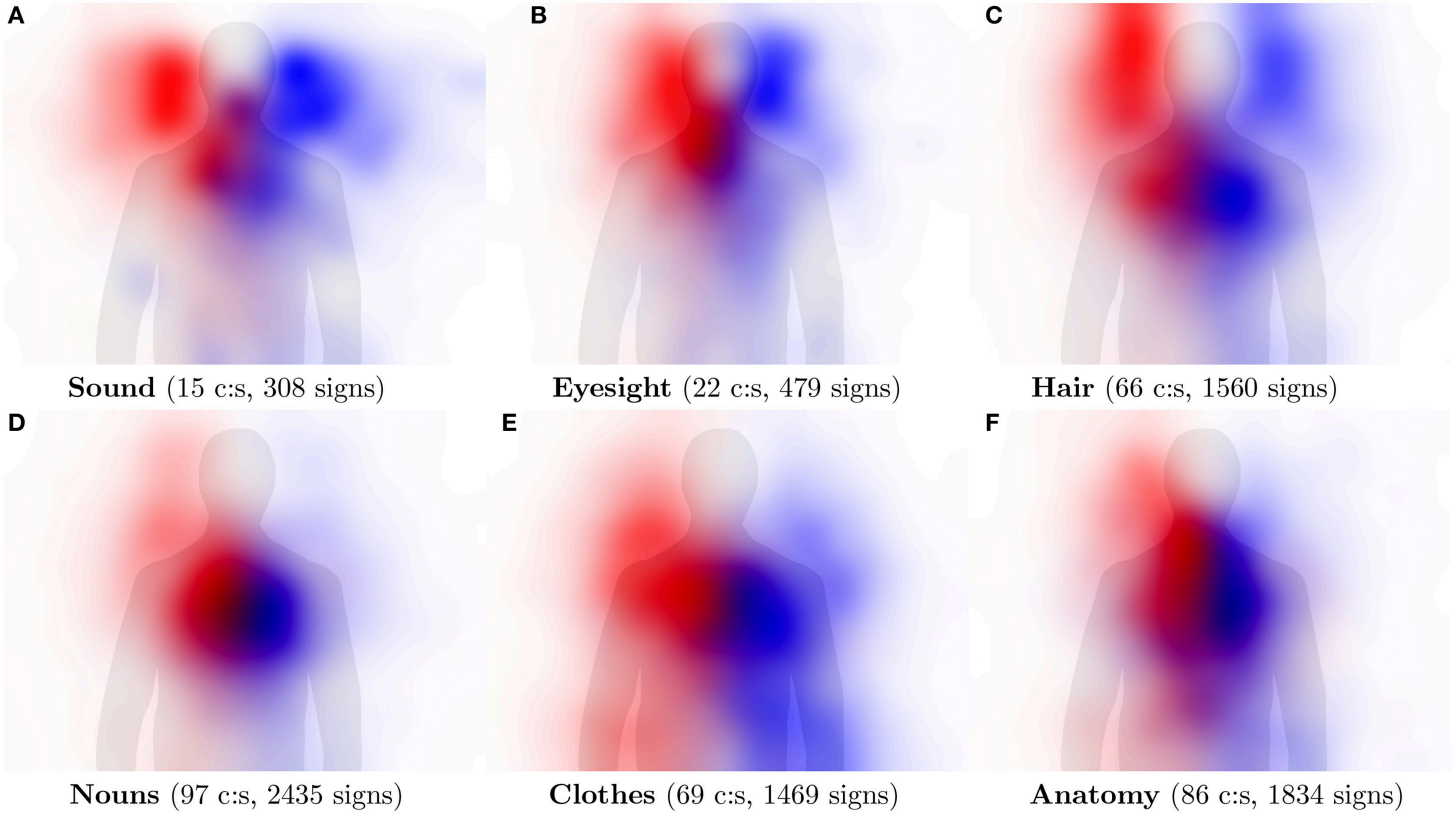
Visualization of hand activity in categories of signs representing a group of concepts (c:s) across multiple languages. The activity of the right hand is shown in red and the left hand in blue.

Rather than looking at individual concepts, Figure 9 shows hand activity over larger categories with hundreds of individual signs each. The *Nouns* category is included as an example of overall hand activity in a semantically *non*-coherent group of concepts, while the remaining pictures in Figure 9 represent semantically (relatively) coherent groups of concepts. Compare the result to Figures 3, 4 in Börstell and Östling (2017, p. 223), who used hand-coded data from Swedish Sign Language for similar categories.

In some cases the categories are strongly associated with particular body parts, which is reflected by our visualizations. For example, *Eyesight* (eyes), *Sound* (ears), and *Hair* (hair) are all clearly associated with the expected body location. In other cases, the categories are associated with large parts of the body, leading to less focused activity patterns, as in the *Clothes* and *Anatomy* categories. Overall, the activity areas are less distinct than for the individual concepts shown in Figure 8, which is expected from the conflation of data from many signs across all languages into one image, but iconic mappings can still be observed.

Besides the figures shown here, we have generated visualizations of sign locations across languages for all individual concepts and sign categories present in the *Spread the Sign* database at the time of download^10^. This may prove useful for other researchers interested in further exploring the cross-linguistic patterns of iconicity with regard to sign locations and semantics.

## 4. Discussion

In this paper, we set out to explore the visual iconicity of signed languages, specifically targeting two phonological parameters—the number of articulators (specifically the number of hands) and the location used in a sign—investigated in the two separate studies presented here. The aim was to explore how these parameters relate to iconicity across languages. Furthermore, since the method of using automated visual processing of large-scale parallel sign language data is a novel one, we also wished to evaluate the accuracy and usefulness of this method, for this and future studies.

### 4.1. Number of articulators

For our first study, we wanted to investigate the distribution of one- vs. two-handed signs across the sign languages in the dataset. Previous studies have suggested that sign language lexicons exhibit quite an even split between one- and two-handed signs (Börstell et al., 2016; Crasborn and Sáfár, 2016; Lepic et al., 2016). Our statistical model (section 3.1.3), using 81 concepts for which we have lexical plurality ratings, indicates that none of the sign languages in our sample has a strong preference for either one- or two-handed signs. Sampling signs corresponding to the concepts in a Swadesh list, which may be used as an approximation of core vocabulary, we see that the distribution corresponds to the one predicted, with an even distribution across all signs and languages. Looking at the individual languages in our data, there is one exception to the overall 50/50 distribution that clearly stands out: Czech Sign Language with 90.1% two-handed signs. This result, along with those of the second and third most two-handed languages (British Sign Language and Russian Sign Language), are likely due to the systematic errors in the automatic processing of those languages described in section 3.1.1. For this reason they have been excluded from the statistical analysis, along with Portuguese Sign Language which had similar problems.

However, when expanding our analysis to a much larger set of concepts, using all concepts encoded by a single word in English to avoid phrases and complicated multisign constructions, we see that the proportion of two-handed signs notably increases to 71% of all signs across languages. Although we do not have an answer to why this is the case, it is possible that less frequent signs are more likely to be two-handed, if one assumes that the the transition from two-handed to one-handed could be a frequency-induced economically motivated reduction (cf. Crasborn and Sáfár, 2016, p. 244). The frequency hypothesis was tested in our statistical model using 81 concepts, but could neither be supported nor conclusively rejected. It may also simply be the case that the sample, though consisting of simplex word forms in English, are encoded by complex, depicting, or multi-sign constructions to a larger extent than the smaller core vocabulary set, as larger lexical databases for individual sign languages—hence implying both frequent and infrequent lexical items included—have previously been found to adhere to the 50/50 pattern, too (see Börstell et al., 2016, p. 393). To what extent the two-handed prominence is a result of misclassification by the algorithm is not known, but manual checks of the classifications have shown mistakes in both directions, without a clear bias in either direction. Nonetheless, the previous claims of a 50/50 distribution are confirmed for a core vocabulary sample, but less so for all languages and concepts in a larger sample of signs.

We do not have a definitive answer as to why the overall distribution of signs exhibits a 50/50 split. It is possible that it has to do with a general preference to organize linguistic units as maximally distinct forms, in a similar fashion to spoken language phoneme inventories preferring distinct (physically and acoustically dispersed) vowels (Lindblom, 1986; Vaux and Samuels, 2015). With this analogy, sign languages would use the one- vs. two-handed division as one main distinction in phonological form, and without taking articulatory economy into account, this could generate an even split. Since we see that at least two-handed forms are associated with certain semantics, we still expect systematicity (and, in our case with plurality, *iconicity*) in some parts of the lexicon, which affects this distribution locally, and further investigations into form–meaning patterns may resolve this issue. As argued for spoken languages (see Dingemanse et al., 2015), there is an advantage in balancing non-arbitrariness and arbitrariness in the word forms of a language, since they have different benefits. For example, whereas the vowel /i/ may be used to iconically denote “smallness,” it would be a major restriction for a language to require such a form–meaning mapping for all uses of /i/—that is, the /i/ would be restricted to a very limited set of words with certain semantics. Similarly, if the one- vs. two-handed division is a useful phonological distinction, it would be a severe limitation to not let that distinction be used arbitrarily. This does, however, not entail that it can *never* be used iconically, and we argue that two-handed forms are often iconically mapped onto plural meaning when possible, but plurality is not a strict requirement when forming two-handed signs.

Concerning the association between lexical plurality and two-handed signs, as found by Börstell et al. (2016), we can say with certainty that this is a valid claim across the languages in our dataset. Our statistical model showed that concepts are significantly more likely to be encoded by a two-handed form if the concept carries plural semantics, even when taking language- and concept-specific variation as well as frequency into account. We argue that this is not only a systematic, but an iconic mapping between plurality and two-handed forms, and an instance of *articulatory plurality* (Börstell et al., 2016). We further corroborate that this is a cross-linguistically valid pattern, and a unique iconic feature of signed language employing the visual modality—unique in the sense of having multiple symmetrical articulators available simultaneously, which adds another articulatory dimension to the previously known mapping between quantity of form and quantity of meaning (Lakoff and Johnson, 1980; Dingemanse et al., 2015). Thus, whereas the more of form is more of content metaphor here limits form to the two hands, the mapping is found in reduplication among spoken languages, and sign languages may also use other multiple articulators for this mapping (e.g., individuated fingers). However, we do not assume the use of multiple articulators in this way to be a unique property of signed language, but rather unique to the visual modality, as it has been shown that hearing non-signers too adhere to articulatory plurality with regard to number of hands when inventing silent gestures for lexically plural concepts (Börstell et al., 2016).

### 4.2. Sign locations

For our second study, we wanted to see to what extent sign locations are employed in similar iconic mappings across languages. In a previous study, Börstell and Östling (2017) showed that it is possible to visualize the location–meaning association of lexical signs in a quantified manner for a single sign language (Swedish Sign Language). By using a series of automated methods for identifying hand activity, including extrapolating hand positions and normalizing sign locations with body pose joint locations as reference points, we were able to visualize areas of hand activity across languages for both individual concepts and larger lexical or semantic categories. We have also demonstrated that these location–meaning associations are iconic, since they correlate strongly with the assignment of iconic locations to concept meanings resulting from an experimental task involving hearing non-signers. Some of these mappings are concretely iconic, such as associating “think” with the (fore)head and “say” with the mouth, and others are metaphorically iconic, such as using the chest/heart area to represent “love.” We thus conclude that both direct and metaphorical mappings contribute to iconic sign formation across sign languages, as argued by Taub (2001). However, we cannot based on our data quantify the extent to which sign language lexicons are iconically motivated, only that the iconic motivation is an available strategy and one that can be verified with quantitative methods.

For the concept categories, the picture becomes more indistinct. This is unsurprising considering it is an overlay of hundreds—or even thousands—of signs corresponding to the concepts in the category, visualized in a single image. Nonetheless, we do see that a category such as *Sound* shows a location pattern similar to that of the individual concept hear—i.e., around the ears—albeit with less distinct boundaries and more noise. Thus, we still consider our method useful in investigating cross-linguistic patterns of systematicity and iconicity in sign locations, which are indeed visible in several cases. It is possible that the visualization across languages *and* concepts simultaneously would show clearer patterns if one were to sample individual concepts based on pre-defined semantic features—that is, rather than by the crude categorizations available in *Spread the Sign*—but it is still likely that some discrepancies and noise will be present. Even though this and a plethora of previous studies have shown that iconicity is a clearly visible feature of signed languages, we would not expect an exact matching of sign locations across languages, even when the location is iconic, seeing as different signs may draw on different iconic mappings (cf. Klima and Bellugi, 1979; Lepic et al., 2016), and thus some variability will appear as noise in this type of visualization. Furthermore, as we argued concerning the one- vs. two-handed division, we would not expect all sign locations to be motivated by meaning, since that could induce unwanted restrictions on possible forms. However, we do acknowledge that many signs are in fact partially iconic, although the iconicity may be found in other parameters than specifically location. Some claims have been made for sign locations exhibiting systematicity rather than iconicity, for instance with signs articulated on the nose or the chin being associated with negative or derogatory meanings in American Sign Language (Frishberg and Gough, 2000, p. 116–118). Thus, we assume that the benefits of balancing arbitrariness and non-arbitrariness are as relevant to signed languages as they are to spoken languages, even if the proportions between the two strategies may differ.

### 4.3. Evaluating the method

The main reason for using automated analysis is efficiency: we can perform several types of analysis on hundreds of thousands of individual signs, in dozens of languages, much faster than any manual coding. This allows large-scale analyses that would be unrealistic to perform manually, and furthermore creates a great potential for exploratory work since working hypotheses can be evaluated in hours rather than months. However, since the computer models available currently perform at less than human performance, there are certain limitations to what we are able to do.

One obvious limitation in using automated classification of sign locations using visual video processing is the fact that this classification only concerns two-dimensional space. The visual modality uses three dimensions, hence any sign location is three-dimensional. Thus, while the automated classification may provide us with height and width information of signs, it does not provide us with any information about the depth of the articulation, such as whether the sign is articulated close to or far away from the signer's body, nor whether the sign has contact with the body part in front of which it is articulated. It is known from previous work on sign iconicity that contact as part of the articulation at a location is in itself meaningful (e.g., Taub, 2001; Meir et al., 2013), but this information is currently unavailable to us. This means, for instance, that signs articulated in neutral space *in front of* the signer are conflated with signs articulated *on* the signer's chest/torso, and whereas the former may lack an iconic mapping between meaning and location, the latter may very well feature such a mapping (e.g., emotion being centered around the signer's chest). It is possible that a model that could single out only signs with body contact articulation would show even stronger patterns of iconicity in different locations.

The above-mentioned limitations are inherent to the video analysis method we use, but we would also like to stress that unexpected systematic and non-systematic errors can be numerous in some cases, even with an automatic system that has a high overall accuracy. For instance, seemingly trivial differences in lightning conditions and how the frame was centered forced us to discard data from four languages. This was discovered only after manually annotating a random subset of signs from each language as one-handed or two-handed. Such an annotation also gives bounds on the rate of non-systematic errors, which should ideally be low compared to the agreement between human raters. We thus strongly encourage researchers to annotate as much data as is practical manually for validation purposes, both to quantify expected problems (when relevant) and to discover unexpected ones. In our case we only considered one variable—the number of active hands—but future work using more fine-grained distinctions, such as handshape and non-manual signals, should ideally contain an evaluation of the accuracy with which each of the variables can be automatically extracted. This is sometimes difficult to do in case the estimated quantity would be complex to annotate, such as in our Study 2 in which we estimate overall levels of hand activity. In these cases, proxy measures such as sign locations may have to be used, although such values are categorical based on phonological assumptions.

Nonetheless, our method here has proven useful in detecting systematic form–meaning mappings across language and concepts even without such an adjustment, showing that automated video processing methods can be useful in analyzing large datasets across languages in the visual modality.

### 4.4. Conclusions

In this paper, we have demonstrated that computational methods may be used in order to detect and quantify patterns of systematicity between form and meaning in the visual language modality. By comparing these patterns to iconicity measurements, we have also been able to show that several of these systematic associations are in fact iconic in nature. For the articulators, we corroborate previous findings, here with a much larger language sample, that lexical plurality of concepts (whether categorical or scalar) correlates with the likelihood of sign languages using a two-handed form: plurality of meaning is iconically mapped onto plural articulators. For sign locations, we see that there are systematic patterns visible across languages, and that these correlate with iconicity ratings given by hearing non-signers, suggesting that concrete and metaphorical iconicity is employed across our sampled sign languages.

Not only do our findings provide further proof of the iconicity potential of language, here specifically the visual iconicity prevalent across languages in the signed modality, but it also adds to a growing body of quantitative linguistics, including research investigating non-arbitrariness across large sets of words and languages (e.g., Urban, 2011; Blasi et al., 2016). We specifically show that the number of hands and the location of signs may be used iconically, across sign languages. We believe that this is one further step toward measuring the prevalence of (non-)arbitrariness in human language, not only across languages but also across modalities, which is an important task in order to explore the fundamental properties that constitute linguistic structure. We also believe that computational methods may become increasingly useful in quantifying language in the visual modality, such as extracting formational features of signs or gestures (e.g., handshapes, movement volume, and manner). To some extent, this has been done previously, but then normally already at the data collection stage, by using technology such as Motion Capture (e.g., Puupponen et al., 2015) or Kinect (e.g., Namboodiripad et al., 2016). Here, we show that even pre-collected video data may be analyzed computationally, in post-production, by employing automated video processing methods.

## Author contributions

The project idea and study design were devised by CB and RÖ (equal contribution). Plurality and location iconicity ratings were collected by CB and RÖ. Sign language data collection and processing were carried out by SC and RÖ (equal contribution), with assistance from CB. The manuscript was drafted by CB and RÖ (equal contribution), and reviewed by SC.

### Conflict of interest statement

The authors declare that the research was conducted in the absence of any commercial or financial relationships that could be construed as a potential conflict of interest.

## Footnotes

^1^Note that visual iconicity is present also in the multimodal communication of speakers of spoken languages, as many co-speech gestures are known to be iconic (Poggi, 2008).

^2^In her paper, other form parameters (e.g., movement) of the sign are also represented as having a double mapping with iconicity and metaphor (Meir, 2010, p. 877).

^3^One-handed signs seem to constitute a higher proportion of sign *tokens* in corpus data, thus the the 50/50 distribution is mainly relevant for sign *types*, i.e., in a dictionary (Crasborn and Sáfár, 2016, p. 244).

^4^The Spread the Sign dictionary has recently been used to create an online database of certain iconic patterns across a sign languages (Kimmelman et al., 2018).

^5^http://www.spreadthesign.com/

^6^*Common Objects in Context* (COCO) is a dataset of 328,000 images of basic objects that have been labeled for developing object recognition models.

^7^We thank one of the reviewers for this suggestion.

^8^Cuban Sign Language was excluded from this manual inspection, since there are < 10 signs each in the one- and two-handed classification group, respectively.

^9^If the four languages with systematic errors are removed, the mean proportion of two-handed signs drops to 53.1%—i.e., even closer to an even distribution.

^10^https://www.ling.su.se/sign-language-iconicity
